# Exploratory echocardiographic strain parameters for the estimation of myocardial infarct size in ST‐elevation myocardial infarction

**DOI:** 10.1002/clc.23608

**Published:** 2021-06-12

**Authors:** Varius Dannenberg, Finn Christiansen, Matthias Schneider, Stefan Kastl, Thomas Martin Hofbauer, Thomas Scherz, Julia Mascherbauer, Dietrich Beitzke, Christoph Testori, Irene Marthe Lang, Andreas Mangold

**Affiliations:** ^1^ Division of Cardiology, Department of Internal Medicine II Medical University of Vienna Vienna Austria; ^2^ Department of Dermatology Landesklinikum Wiener Neustadt Wiener Neustadt Austria; ^3^ Department of Internal Medicine Karl Landsteiner University of Health Sciences, University Hospital St. Poelten Krems Austria; ^4^ Department of Biomedical Imaging and Image‐guided therapy Medical University of Vienna Vienna Austria; ^5^ Department of Internal Medicine, Cardiology and Nephrology Landesklinikum Wiener Neustadt Vienna Austria; ^6^ Department of Emergency Medicine Medical University of Vienna Vienna Austria

## Abstract

**Background:**

Outcome after ST‐elevation myocardial infarction (STEMI) can be most reliably estimated by cardiac magnetic resonance (CMR) imaging. However, CMR is expensive, laborious, and has only limited availability. In comparison, transthoracic echocardiography (TTE) is widely available and cost‐efficient.

**Hypothesis:**

TTE strain parameters can be used as surrogate markers for CMR‐measured parameters after STEMI.

**Methods:**

TTE strain analysis was performed of patients included in a controlled, prospective STEMI trial (NCT01777750) 4 ± 2 days after the event. Longitudinal peak strain (LPS), post‐systolic shortening, early systolic lengthening, early systolic lengthening time, and time to peak shortening were measured, and index parameters were computed. Global longitudinal strain (GLS) and ejection fraction (EF) were compiled. Parameters were correlated with CMR‐measured variables 4 ± 2 days after STEMI.

**Results:**

In 70 STEMI patients, high quality CMR and TTE data were available. Highest correlation with CMR‐measured infarct size was observed with GLS (r = 0.577, p < 0.0001), LPS (r = 0.571, p < 0.0001), and EF (r = −0.533, p < 0.0001). Highest correlation with CMR‐measured area at risk was observed with GLS (r = 0.666, p < 0.0001), LPS (0.661, p < 0.0001) and early systolic lengthening index (r = 0.540, p < 0.0001). Receiver operating characteristics for the detection of large infarcts (quartile with highest infarct size) showed the highest area under the curve for LPS, GLS, EF, and myocardial dysfunction index. Multiple linear regression displayed the best association between GLS and infarct size.

**Conclusion:**

Exploratory strain parameters significantly correlate with CMR‐measured area at risk and infarct size and are of potential interest as endpoint variables in clinical trials.

**Abbreviations**
AAR: area at risk
AUC: area under the curve
AVC: aortic valve closure
CMR: cardiac magnetic resonance imaging
ELI: early systolic lengthening index
ELT: early systolic lengthening time
ESL: early systolic lengthening
GLS: global longitudinal strain
IQR: interquartile range
LGE: late gadolinium‐enhanced
LPS: longitudinal peak strain
MDI: myocardial dysfunction index
MSI: myocardial salvage index
MTH: mild therapeutic hypothermia
MVO: microvascular obstruction
pPCI: primary percutaneous coronary intervention
PSI: post‐systolic shortening index
PSS: post‐systolic shortening
ROC: receiver operating characteristics
SD: standard deviation
STATIM: strategic targeted temperature in myocardial infarction
STEMI: ST‐elevation myocardial infarction
TTE: transthoracic echocardiography
TTP: time to peak shortening

## 1 INTRODUCTION

Cardiovascular disease is the leading cause of death in industrial societies,^1^ with high morbidity after ST‐elevation myocardial infarction (STEMI).^2^ Trials testing novel therapeutic strategies to alleviate myocardial damage beyond primary percutaneous coronary intervention (pPCI) are still of interest.^3^ Cardiac magnetic resonance (CMR) is the gold standard to determine cardiac damage and function after STEMI and is therefore employed for primary endpoints in clinical trials.^4^ It is still controversial which CMR parameter should be used for optimal estimation of myocardium salvaged by cardioprotective therapies.^5^ Measuring edema‐based area at risk (AAR) and calculating the myocardial salvage index (MSI) have been proposed^6^ but were lately called into doubt.^5^ Currently, CMR‐measured infarct size and microvascular obstruction (MVO) proved to be the best predictors for outcome in STEMI patients.^7^ A meta‐analysis of 10 randomized trials showed a 7‐fold higher incidence of heart failure hospitalizations and all‐cause mortality in patients in the quartile with the highest infarct size compared with patients in the quartile with the smallest infarct size.^8^ However, CMR is expensive, laborious, and has only limited availability, which complicates interventional studies and limits participating centres. In comparison, transthoracic echocardiography (TTE) is widely available, cost‐efficient, and practicable at the bedside. Limited sensitivity and a substantial observer bias are major disadvantages of the technique. Correct assessment of wall motion is associated with significant inter‐ and intraobserver variability, even if performed by experienced echocardiographers.^9^ In the past, superiority in interstudy reproducibility of CMR compared to 2D TTE was shown.^10^ Infarct size cannot be measured by TTE, but functional parameters reflecting necrotic segments and stunned myocardium can be measured. Strain analysis applying speckle tracking in TTE images quantifies deformation of myocardial segments. It is an algorithm‐based technique, which reduces investigator bias and is already used in clinical routine to estimate cardiac function. Global longitudinal strain (GLS) has become an established parameter. Changes in GLS are related to ischemic regions as validated by contrast‐enhanced CMR.^11^ Other strain parameters, such as post‐systolic shortening (PSS) and post‐systolic shortening index (PSI), were predictive of heart failure in patients after STEMI.^12^ Post‐systolic shortening and wall thickening are known as parameters reflecting ischemia and short‐term hibernation.^13^, ^14^ Further parameters of interest are early systolic lengthening (ESL), early systolic lengthening index (ELI), and myocardial dysfunction index (MDI).^15^, ^16^ Improved ultrasound technology allows to evaluate TTE variables as potential surrogate endpoints in clinical trials. Furthermore, novel strain measurements might be useful in routine patient care as prognostic and surveillance parameters.

In the present study, we aimed to assess 2D strain parameters as surrogate markers compared to CMR‐measured parameters in STEMI patients who were included in a controlled, prospective study.

## 2 METHODS

### 2.1 Patients

Patients included in the present study were participants of the controlled, randomized, prospective *strategic targeted temperature in myocardial infarction* (STATIM) trial, in which mild therapeutic hypothermia (MTH) was compared with standard care regarding AAR after STEMI. The primary endpoint was MSI as measured by CMR.^5^ All primary and secondary endpoints were negative.^17^ STEMI patients between 18 and 75 years treated with pPCI in the cath lab of the Vienna General Hospital between 2013 and 2016 were included (*n* = 101). Major inclusion criteria were anterior or inferior infarction, significant ST‐segment elevation, and <6 h symptom duration. Major exclusion criteria were cardiac arrest, history of acute coronary syndrome, coronary artery stenting or bypass grafting, chronic or acute heart failure, thrombolysis, infection, end‐stage kidney, hepatic or pulmonary disease, recent stroke, and being a female of childbearing age. The STATIM trial was registered under (NCT01777750). All study participants gave written informed consent prior to the inclusion under approval of the Ethics Committee of the Medical University of Vienna, Austria (approval reference number 1497/2012). All investigations were performed in accordance with the Declaration of Helsinki. 70 of 101 patients had both a high‐quality CMR and echocardiography investigation within 4 ± 2 days after pPCI and were included in the present study.

### 2.2 Cardiac magnetic resonance

**FIGURE 1:**
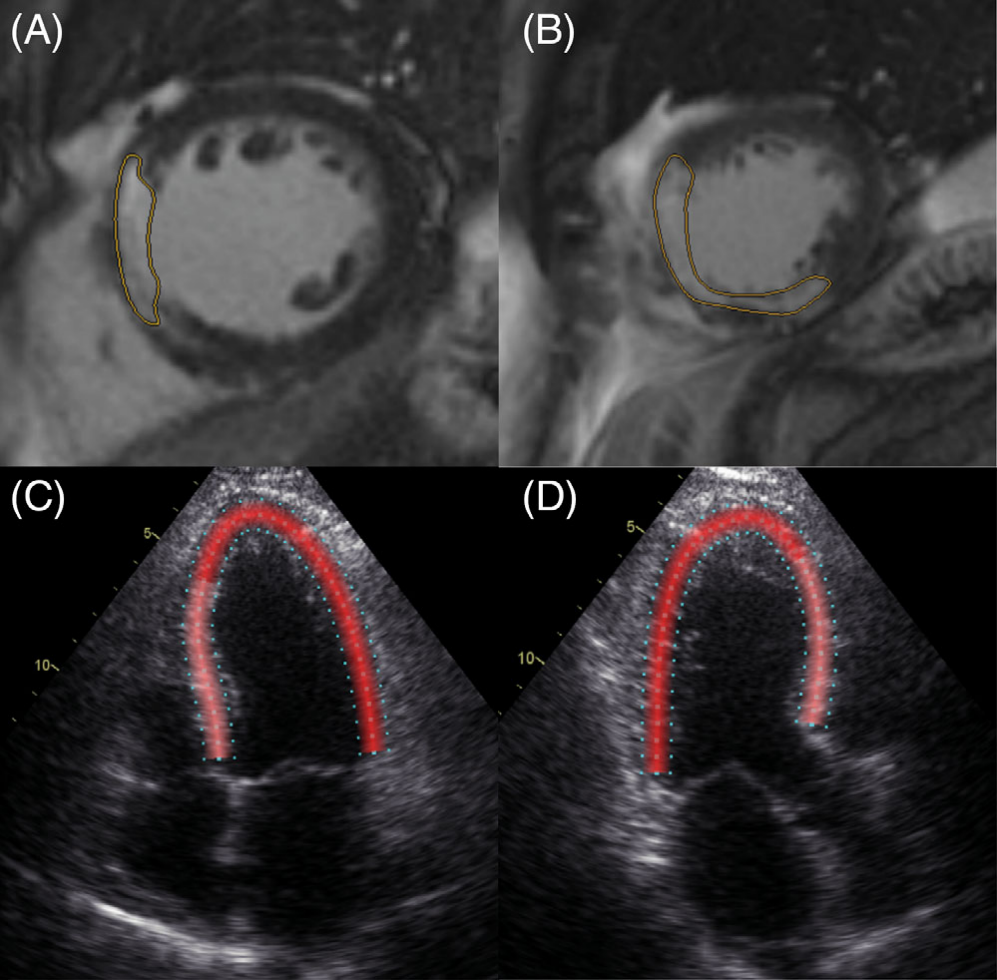
Corresponding CMR and TTE imaging. Infarcted area in two short‐axis slices by CMR (A, B). Corresponding speckle tracking analysis in a four‐chamber (C) and three‐chamber view (D) by TTE

Cardiac magnetic resonance (CMR) was described in detail before.^17^ The volume of the entire left ventricular myocardium was assessed. Infarct size was measured 4 ± 2 days after pPCI using a 1.5 T system (Avanto Fit, Siemens Healthineers) delineating late gadolinium‐enhanced (LGE) myocardium in short‐axis views (Figure 1, Panel A, B). The percentage of infarcted area relative to total myocardium was calculated. In addition, the edema‐based AAR, by T1 and T2 mapping, and MVO, delineated as hypoenhanced infarct core area in LGE studies were measured. CMR was conducted using standardized protocols.^18^ For late gadolinium enhancement, 0.15 ml/kg gadobutrol (0.1 mmoL/ml, Gadovist, Bayer) was administered. Left ventricular volumes and ejection fraction (EF) were assessed. Patients were divided into quartiles according to infarct size. A cardiovascular radiologist processed and evaluated the images using the QMass postprocessing software package (Medis Medical Imaging).

### 2.3 Echocardiography

**FIGURE 2:**
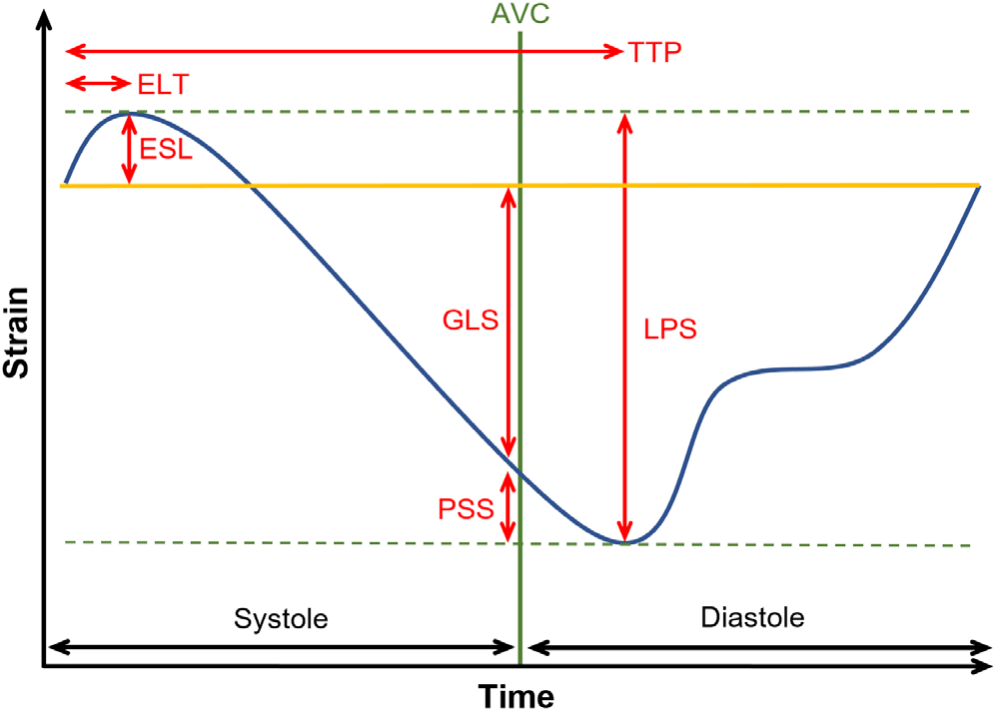
Schematic explanation of strain parameters. AVC, aortic valve closure; ESL, early systolic lengthening; ELT, early systolic lengthening time; LPS, longitudinal peak systolic strain; GLS, global longitudinal strain; PSS, post‐systolic shortening; TTP, time to peak shortening

Echocardiographic studies were performed by experienced observers on a GE Vivid E9 4 ± 2 days after pPCI. Speckle tracking analysis was performed using specific software (Figure 1, Panel C, D, EchoPacs, GE Healthcare). The following values were measured in each segment of the left ventricular myocardium according to the 18 segments model^19^: longitudinal peak strain (LPS), PSS, ESL, early systolic lengthening time (ELT), and time to peak shortening (TTP). LPS is the maximum strain within the entire cardiac cycle. Post‐systolic shortening is defined as the maximum shortening after aortic valve closure (AVC). ESL is the maximum lengthening between the start of the cardiac cycle, defined as the beginning of the QRS‐complex, and the AVC. Early systolic lengthening time is the time from the start of the cardiac cycle to the ESL. Time‐to‐peak shortening is the time from the beginning of the cardiac cycle to the maximum overall shortening (Figure 2). Using these measurements, the following index parameters were computed: post‐systolic shortening index ([PSS/LPS)]*100), ELI, (ESL amplitude/LPS]*100), and MDI ([(ESL amplitude + PSS amplitude)/LPS]*100). Values were averaged by dividing the cumulative values of every parameter by the number of tracked segments. In addition, the GLS, defined as the highest average strain at one point in time during systole, was obtained. It is noteworthy that, according to a consensus document for 2D strain values, we describe a decrease in GLS and LPS as a trend towards zero, despite negative values.^20^

### 2.4 Statistical analysis

Baseline characteristics are displayed using descriptive statistics. All parameters were tested for normal distribution applying histograms complemented by Shapiro–Wilk tests and Kolmogorov–Smirnov tests. In case of parametric distribution, data are given as mean ± standard deviation (SD); in case of non‐parametric distribution, data are given as median and interquartile range (IQR). Pearson correlations were applied, including all echocardiographic parameters, AAR, and MSI, due to normal distribution. Spearman correlations were applied including all echocardiographic parameters, MVO and infarct size due to non‐parametric distribution. A p‐value below 0.05 was considered significant. Receiver operating characteristics (ROC) curves were calculated for all echocardiographic parameters to detect patients in the quartile with the highest infarct size. All areas under the curves (AUC) and the Youden indices were calculated.^21^ Bonferroni‐Holm correction was performed for all correlations and ROC curves. Multiple linear regression was performed using a stepwise approach. All parameters were inserted in the model as independent variables with infarct size as the dependent variable. For statistical analysis, SPSS (Version 27, SPSS Inc.) was used.

## 3 RESULTS

### 3.1 Patients

Patients, from whom high quality CMR and TTE data were available, were included in the analysis (*n* = 70). Mean age was 56 ± 10 years, the majority of patients were male (19% female). Detailed baseline characteristics are displayed in Table 1.

**TABLE 1:** Patient characteristics. Data are displayed as n (%), median [IQR], or as mean ± SD

Characteristic	Parameters (*n* = 70)
Age, years	56 ± 10
Female sex, *n*	13 (19)
Weight, kg	85 ± 16
Hypertension, *n*	24 (34)
Diabetes, *n*	7 (10)
Dyslipidaemia, *n*	17 (24)
Current smoker, *n*	39 (56)
Familial history of CAD, *n*	18 (26)
Obesity, *n*	19 (27)
Body mass index, kg/m^2^	28 ± 4.6
Sinus rhythm, *n*	66 (94)
Creatinine, μmol/l	0.9 ± 0.3
Infarct‐related artery	
Left anterior descending artery, *n*	36 (51)
Circumflex artery, *n*	6 (8.6)
Right coronary artery, *n*	28 (40)
Multivessel disease, *n*	40 (57)
Cardiac magnetic resonance imaging	
EF, %	51 ± 12
MVO, ml	1 [0–3]
Left ventricular volume, ml	135 ± 39
AAR, ml	44 ± 23
AAR, %	33 [26–489]
Infarct size volume, ml	29 ± 22
Infarct size volume, %	20 [1.3–31.1]
Quartile highest infarct size volume, %	43.6 [36.4–48.4]
Echocardiography	
EF, %	47 ± 9
GLS, %	−12.4 ± 4.2
LPS, %	−13.2 ± 4.0
MDI, %	21.8 [13.1–30.7]
PSI, %	10.9 [6.6–19.19]
PSS, %	−1.8 [−2.4 ‐ ‐1.0]
ELI, %	7.8 [5.0–15.5]
ESL, %	0.88 [5.4–1.24]
ELT, msec	51 [34–75]
TTP, msec	398 [371–437]

Abbreviations: AAR, area at risk; CAD, coronary artery disease; EF, Ejection fraction; ELI, early systolic lengthening index; ELT, early systolic lengthening time; ESL, early systolic lengthening; GLS, global longitudinal strain; IQR, interquartile range; LPS, longitudinal peak strain; MDI, myocardial dysfunction index; MVO, microvascular obstruction; PSI, post‐systolic shortening index; PSS, post‐systolic shortening; SD, standard deviation; TTP, time‐to‐peak shortening.

### 3.2 TTE‐measured strain parameters correlate with CMR‐measured infarct size and AAR

**FIGURE 3:**
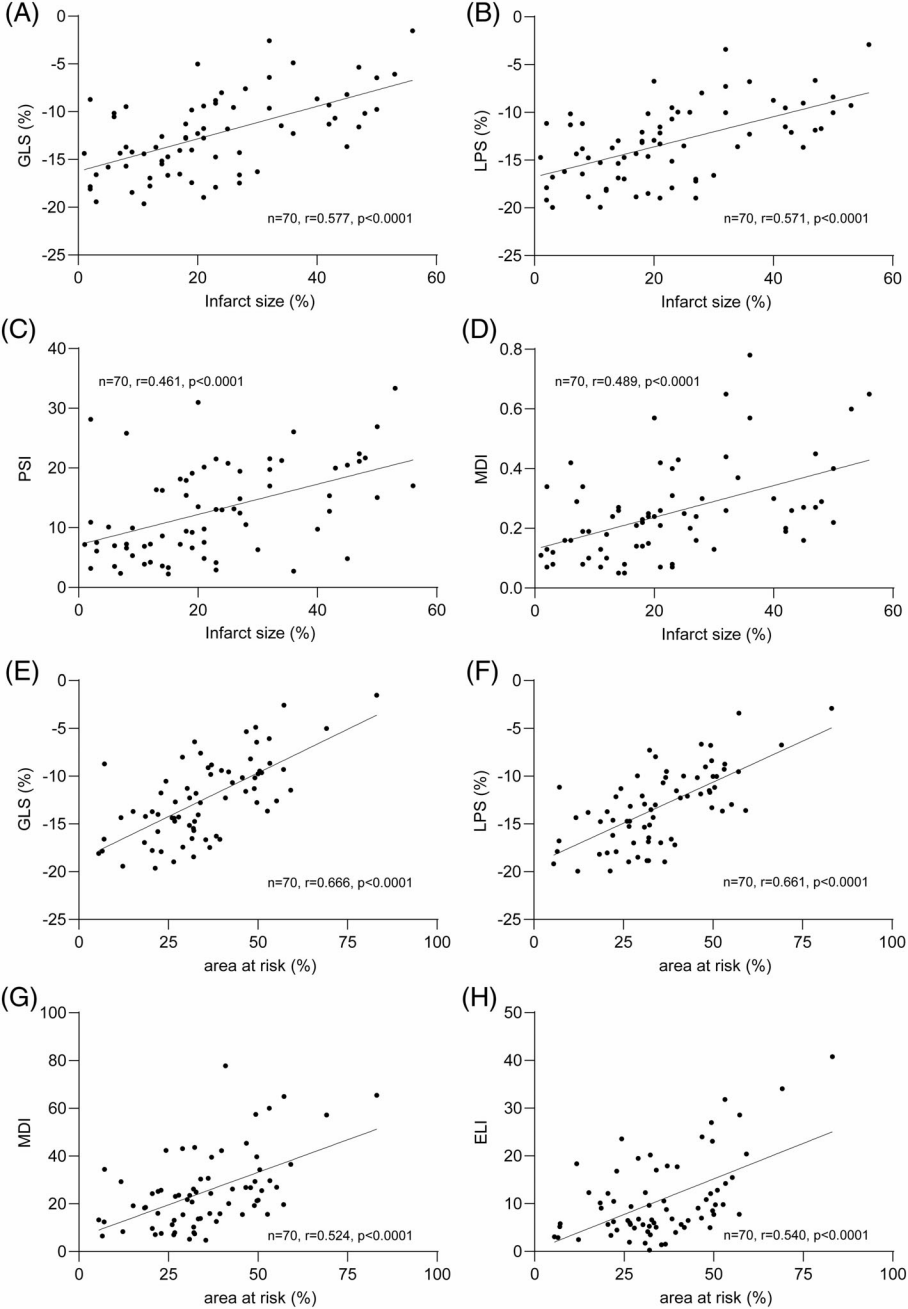
Correlations between TTE and CMR parameters. GLS, global longitudinal strain; LPS, longitudinal peak strain; PSI, post‐systolic shortening index; MDI, myocardial dysfunction index; ELI, early systolic lengthening index

Significant correlations with infarct size were observed for GLS (r = 0.577, p < 0.0001), LPS (r = 0.571, p < 0.0001), EF (r = −0.533, p < 0.0001), MDI (r = 0.489, p < 0.0001), PSI (r = 0.461, p < 0.0001), ELI (r = 0.387, p = 0.005), and TTP (r = 0.248, p = 0.038). All other parameters did not correlate significantly (Table S1, Figure 3).

**FIGURE 4:**
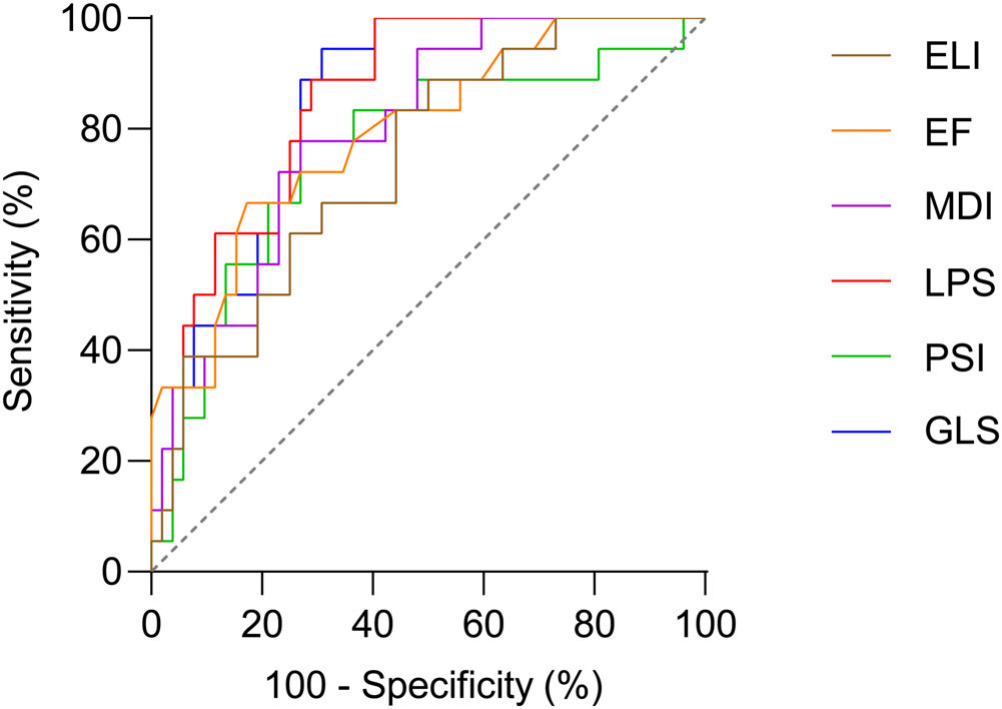
Receiver‐operating‐characteristics curves. EF, ejection fraction; ELI, early systolic lengthening index; ELT, early systolic lengthening time; ESL, early systolic lengthening; GLS, global longitudinal strain; LPS, longitudinal peak strain; MDI, myocardial dysfunction index; PSI, post‐systolic shortening index; PSS, post‐systolic shortening; TTP, time to peak shortening

ROC curves were calculated for echo parameters to detect the quartile with the highest infarct size. Significant AUCs were observed for LPS (0.84 [0.74–0.93], p < 0.0001), GLS (0.83 [0.74–0.93], p < 0.0001), EF (0.80 [0.68–0.91], p < 0.0001), MDI (0.79 [0.68–0.90], p < 0.0001), PSI (0.76 [0.62–0.89], p = 0.006) and ELI (0.73 [0.60–0.86], p = 0.014). Complete data are provided in Table S5. Significant AUCs are displayed in Figure 4.

A multiple linear regression model applying a stepwise approach revealed a significant association between EF, GLS, and infarct size (F [2, 64] = 23.18, p < 0.0001). A decrease of the GLS by 1.44% (p < 0.0001) and a decrease of the EF by 0.38% (p < 0.05) were associated with an increase of the infarct size by 1%.

Furthermore, we calculated correlations with AAR, MVO, and MSI. Interestingly, strong correlations were observed between AAR and GLS (r = 0.666, p < 0.0001), LPS (r = 0.661, p < 0.0001), EF (r = −0.443, p = 0.0006), MDI (r = 0.524, p < 0.0001), PSI (r = 0.469, p = 0.0003 and ELI (r = 0.540, p < 0.0001). Other CMR‐measured variables and echo parameters resulted in weaker correlations. Complete data are provided in the Tables S1–S4.

## 4 DISCUSSION

In the present study, we evaluated a broad set of echocardiographic strain parameters as outcome variables after STEMI compared to CMR‐measured parameters. This data set may build a basis for further evaluation of strain parameters as surrogate marker endpoints in STEMI intervention trials.

The study cohort of the present study were participants of a prospective STEMI trial, in which MTH was tested as an adjunctive therapy. In line with CMR endpoint results, we observed no differences in echocardiographic measurements between the treatment groups (data not shown).

Strain analysis in TTE has evolved rapidly in the past 10 years,^20^ and GLS is broadly used to assess left ventricular function in daily routine. Recently, it was shown that GLS has superior prognostic value over EF measurements in heart failure.^22^ Moreover, strain analysis algorithms provide a large data set beyond GLS. The amount of shortening for every segment at every time point during the cardiac cycle is displayed. Dyssynchronous contraction patterns, shortening during diastole, or lengthening during systole can be observed. Lengthening at the beginning of systole, the ESL, is displayed in percent of the overall length of the myocardium for each acoustic window.^23^ The corresponding index (ELI) can be interpreted as the amount of lengthening in relation to the overall shortening in percent. Shortening of the myocardium after aortic valve closure (PSS) has already been recognized as a sensitive parameter in coronary artery disease several years ago.^24^ The corresponding index (PSI) can be interpreted as the amount of shortening occurring after aortic valve closure in relation to the overall shortening in percent. The MDI, which combines ESL and PSS and describing the proportion of 'wasted work', was recently introduced.^16^ This wasted work impacts negatively on cardiac output and must be compensated by the remaining myocardium. The mechanical interaction between infarct, border, and remote normal zone leads to an impaired myocardial performance with dyssynchronous contraction of the different segments.^25^ Furthermore, ELT and TTP were investigated in patients with ischemic heart disease.^15^, ^26^

Infarct size after STEMI measured by CMR is a predictor for mortality and hospitalization for heart failure. In a recent meta‐analysis of patients with myocardial infarction, an infarct size of >29.8% (highest quartile) resulted in all‐cause mortality of 3.8%, whereas an infarct size of <8.0% (lowest quartile) resulted in all‐cause mortality of only 0.9% (overall p = 0.002).^8^ Still, the delineation of LGE myocardium for the estimation of infarct size does not differentiate between a large endocardial and a smaller transmural infarction, although differences in outcome are suggested.^27^ The correlation between infarct size and strain parameters in our study is overall strong, but established values, such as EF, can compete.

ROC analysis for the highest quartile of infarct size showed an AUC of 0.83 and 0.84 for GLS and LPS, respectively, which corresponds to a very good diagnostic accuracy. Weighting sensitivity and specificity equally (Youden index), sensitivity of the GLS is 94%, while it is only 67% for the EF. Still, the EF has a high specificity of 83% and an AUC of 0.80, which is also in the range of a very good diagnostic modality. Furthermore, the MDI, PSI, and ELI displayed AUCs between 0.73 and 0.79, which can be rated as good diagnostic accuracy. Interestingly, the PSI and the MDI displayed lower sensitivity than the GLS or the LPS but higher specificity. Therefore, a two‐step approach might be useful, in which PSI and MDI are measured only after the GLS displays decreased strain. Region‐specific changes in strain parameters might also be of potential interest in patients with ischemic heart disease.

The multiple linear regression favored the GLS and the EF as the most accurate diagnostic variables in the present cohort for global estimation of the infarct size, supporting their use in routine patient care. A recent study observed that a 1.27% decrease in GLS was associated with a 1% increase in infarct size, consistent with the results of our multiple linear regression model.^28^

Significant correlations of GLS, LPS, EF, MDI, PSI, ELI were also observed with CMR‐measured AAR (Table S2). These results underscore the hypothesis that adverse myocardial motion quantified by strain analysis in TTE reflect ischemic and/or stunned myocardium quantified as AAR in CMR.^13^ Thus, beneficial effects of cardioprotective therapies could well be monitored by TTE‐based strain measurements.

### 4.1 Limitations

The present analysis was not a pre‐specified endpoint of the STATIM trial. High‐quality echocardiographic data and CMR data were not available from every patient included in the STATIM trial, thereby limiting the final sample size.

### 4.2 Conclusion

Exploratory strain parameters and established 2D echocardiographic parameters are significantly correlated with CMR‐measured AAR and infarct size. Strain parameters should be tested as secondary endpoints in prospective trials to evaluate their suitability as surrogate markers for outcome after STEMI.

## CONFLICT OF INTEREST

The authors declare no potential conflict of interest.

## Supporting information


**Table S1** Correlation of TTE parameters with CMR‐measured infarct size. EF, ejection fraction; GLS, global longitudinal peak systolic strain; LPS, longitudinal peak strain; MDI, myocardial dysfunction index; PSI, post‐systolic shortening index; PSS, post‐systolic shortening; ELI, early systolic lengthening index; ESL, early systolic lengthening; TTP, time‐to‐peak shortening; ELT, early systolic lengthening time.
**Table S2:** Correlation of TTE parameters with CMR‐measured area at risk. EF, ejection fraction; GLS, global longitudinal peak systolic strain; LPS, longitudinal peak strain; MDI, myocardial dysfunction index; PSI, post‐systolic shortening index; PSS, post‐systolic shortening; ELI, early systolic lengthening index; ESL, early systolic lengthening; TTP, time‐to‐peak shortening; ELT, early systolic lengthening time.
**Table S3:** Correlation of TTE parameters with CMR‐measured myocardial salvage index. EF, ejection fraction; GLS, global longitudinal peak systolic strain; LPS, longitudinal peak strain; MDI, myocardial dysfunction index; PSI, post‐systolic shortening index; PSS, post‐systolic shortening; ELI, early systolic lengthening index; ESL, early systolic lengthening; TTP, time‐to‐peak shortening; ELT, early systolic lengthening time.
**Table S4:** Correlation of TTE parameters with CMR‐measured microvascular obstruction. EF, ejection fraction; GLS, global longitudinal peak systolic strain; LPS, longitudinal peak strain; MDI, myocardial dysfunction index; PSI, post‐systolic shortening index; PSS, post‐systolic shortening; ELI, early systolic lengthening index; ESL, early systolic lengthening; TTP, time‐to‐peak shortening; ELT, early systolic lengthening time.
**Table S5:** Receiver‐operating characteristics for the detection of patients with large infarcts (quartile with highest infarct size). AUC, area under the curve; EF, ejection fraction; GLS, global longitudinal peak systolic strain; LPS, longitudinal peak strain; MDI, myocardial dysfunction index; PSI, post‐systolic shortening index; PSS, post‐systolic shortening; ELI, early systolic lengthening index; ESL, early systolic lengthening; TTP, time‐to‐peak shortening; ELT, early systolic lengthening time.

## Data Availability

All data will be made available upon request
