# Supplementary material for: Exploratory echocardiographic strain parameters for the estimation of myocardial infarct size in ST‐elevation myocardial infarction
Source: Clin Cardiol. 2021 Jun 12;44(7):925–31. doi: 10.1002/clc.23608 (PMC8259148; doi:10.1002/clc.23608)
Supplement: Supplementary file 1 — Table S1 Correlation of TTE parameters with CMR‐measured infarct size. EF, ejection fraction; GLS, global longitudinal peak systolic strain; LPS, longitudinal peak strain; MDI, myocardial dysfunction index; PSI, post‐systolic shortening index; PSS, post‐systolic shortening; ELI, early systolic lengthening index; ESL, early systolic lengthening; TTP, time‐to‐peak shortening; ELT, early systolic lengthening time. Table S2: Correlation of TTE parameters with CMR‐measured area at risk. EF, ejection fraction; GLS, global longitudinal peak systolic strain; LPS, longitudinal peak strain; MDI, myocardial dysfunction index; PSI, post‐systolic shortening index; PSS, post‐systolic shortening; ELI, early systolic lengthening index; ESL, early systolic lengthening; TTP, time‐to‐peak shortening; ELT, early systolic lengthening time. Table S3: Correlation of TTE parameters with CMR‐measured myocardial salvage index. EF, ejection fraction; GLS, global longitudinal peak systolic strain; LPS, longitudinal peak strain; MDI, myocardial dysfunction index; PSI, post‐systolic shortening index; PSS, post‐systolic shortening; ELI, early systolic lengthening index; ESL, early systolic lengthening; TTP, time‐to‐peak shortening; ELT, early systolic lengthening time. Table S4: Correlation of TTE parameters with CMR‐measured microvascular obstruction. EF, ejection fraction; GLS, global longitudinal peak systolic strain; LPS, longitudinal peak strain; MDI, myocardial dysfunction index; PSI, post‐systolic shortening index; PSS, post‐systolic shortening; ELI, early systolic lengthening index; ESL, early systolic lengthening; TTP, time‐to‐peak shortening; ELT, early systolic lengthening time. Table S5: Receiver‐operating characteristics for the detection of patients with large infarcts (quartile with highest infarct size). AUC, area under the curve; EF, ejection fraction; GLS, global longitudinal peak systolic strain; LPS, longitudinal peak strain; MDI, myocardial dysfunction index; PSI, post‐systol [file CLC-44-925-s001.docx]

**Supplemental Material**

**Exploratory echocardiographic strain parameters for the estimation of myocardial infarct size in STEMI**

Varius Dannenberg^1^, Finn Christiansen^1^, Matthias Schneider^1^, Stefan Kastl^1^, Thomas Martin Hofbauer^1^, Thomas Scherz^1,2^, Julia Mascherbauer^1^, Dietrich Beitzke^3^, Christoph Testori^4, 5^, Irene Marthe Lang^1^, Andreas Mangold^1^

^1^Division of Cardiology, Department of Internal Medicine II, Medical University of Vienna, Vienna, Austria.

^2^Department of Dermatology, Landesklinikum Wiener Neustadt, Austria

^3^Department of Biomedical Imaging and Image-guided therapy, Medical University of Vienna, Vienna, Austria.

^4^Department of Internal Medicine, Cardiology and Nephrology, Landesklinikum Wiener Neustadt, Austria

^5^Department of Emergency Medicine, Medical University of Vienna, Austria

Correspondence to:

Andreas Mangold, MD, PhD; Department of Internal Medicine II, Division of Cardiology, Medical University of Vienna; Waehringer Guertel 18-20, Austria, A-1090 Vienna;

Tel.: +43 1 40400 46140; Fax: +43 1 40400 46120; [andreas.mangold@meduniwien.ac.at](mailto:andreas.mangold@meduniwien.ac.at).

**Supplemental Tables**

|  | **n** | **r** | **p-value** |
| --- | --- | --- | --- |
| GLS | 70 | 0.577 | <0.0001 |
| LPS | 70 | 0.571 | <0.0001 |
| EF | 70 | -0.533 | <0.0001 |
| MDI | 70 | 0.489 | <0.0001 |
| PSI | 70 | 0.461 | <0.0001 |
| ELI | 70 | 0.387 | 0.005 |
| TTP | 70 | 0.248 | 0.038 |
| PSS | 70 | 0.262 | 0.116 |
| ELT | 70 | 0.254 | 0.102 |
| ESL | 70 | 0.088 | 0.466 |

**Supplemental Table 1: Correlation of TTE parameters with CMR-measured infarct size**. EF, ejection fraction; GLS, global longitudinal peak systolic strain; LPS, longitudinal peak strain; MDI, myocardial dysfunction index; PSI, post-systolic shortening index; PSS, post-systolic shortening; ELI, early systolic lengthening index; ESL, early systolic lengthening; TTP, time-to-peak shortening; ELT, early systolic lengthening time.

|  | **n** | **r** | **p-value** |
| --- | --- | --- | --- |
| GLS | 70 | 0.666 | <0.0001 |
| LPS | 70 | 0.661 | <0.0001 |
| EF | 70 | -0.443 | 0.0006 |
| ELI | 70 | 0.540 | <0.0001 |
| MDI | 70 | 0.524 | <0.0001 |
| PSI | 70 | 0.469 | 0.0003 |
| ELT | 70 | 0.295 | 0.052 |
| PSS | 70 | 0.282 | 0.054 |
| ESL | 70 | 0.179 | 0.277 |
| TTP | 70 | -0.038 | 0.756 |

**Supplemental Table 2: Correlation of TTE parameters with CMR-measured area at risk**. EF, ejection fraction; GLS, global longitudinal peak systolic strain; LPS, longitudinal peak strain; MDI, myocardial dysfunction index; PSI, post-systolic shortening index; PSS, post-systolic shortening; ELI, early systolic lengthening index; ESL, early systolic lengthening; TTP, time-to-peak shortening; ELT, early systolic lengthening time.

|  | **n** | **r** | **p-value** |
| --- | --- | --- | --- |
| EF | 70 | 0.427 | 0.002 |
| GLS | 70 | -0.285 | 0.150 |
| LPS | 70 | -0.275 | 0.170 |
| MDI | 70 | -0.262 | 0.198 |
| PSI | 70 | -0.256 | 0.197 |
| ELI | 70 | -0.183 | 0.647 |
| PSS | 70 | -0.079 | 1 |
| TTP | 70 | -0.070 | 1 |
| ESL | 70 | -0.034 | 1 |
| ELT | 70 | -0.033 | 0.788 |

**Supplemental table 3: Correlation of TTE parameters with CMR-measured myocardial salvage index.** EF, ejection fraction; GLS, global longitudinal peak systolic strain; LPS, longitudinal peak strain; MDI, myocardial dysfunction index; PSI, post-systolic shortening index; PSS, post-systolic shortening; ELI, early systolic lengthening index; ESL, early systolic lengthening; TTP, time-to-peak shortening; ELT, early systolic lengthening time.

|  | **n** | **r** | **p-value** |
| --- | --- | --- | --- |
| EF | 70 | -0.435 | 0.002 |
| PSI | 70 | 0.240 | 0.405 |
| LPS | 70 | 0.223 | 0.511 |
| GLS | 70 | 0.217 | 0.496 |
| MDI | 70 | 0.206 | 0.525 |
| ELI | 70 | 0.138 | 1 |
| ELT | 70 | 0.109 | 1 |
| PSS | 70 | 0.109 | 1 |
| TTP | 70 | 0.107 | 0.754 |
| ESL | 70 | -0.011 | 0.929 |

**Supplemental table 4: Correlation of TTE parameters with CMR-measured microvascular obstruction.** EF, ejection fraction; GLS, global longitudinal peak systolic strain; LPS, longitudinal peak strain; MDI, myocardial dysfunction index; PSI, post-systolic shortening index; PSS, post-systolic shortening; ELI, early systolic lengthening index; ESL, early systolic lengthening; TTP, time-to-peak shortening; ELT, early systolic lengthening time.

|  | **AUC (95% CI)** | **p-value** | **best cut-off** | **sensitivity** | **specificity** | **accuracy** |
| --- | --- | --- | --- | --- | --- | --- |
| LPS, % | 0.84 (0.74 − 0.93) | <0.0001 | -12.3 | 94% | 69% | 74% |
| GLS, % | 0.83 (0.74 − 0.93) | <0.0001 | -12.6 | 89% | 71% | 76% |
| EF, % | 0.80 (0.68 − 0.91) | <0.0001 | 54 | 67% | 83% | 79% |
| MDI, % | 0.79 (0.68 − 0.90) | <0.0001 | 25.4 | 78% | 73% | 74% |
| PSI, % | 0.76 (0.62 − 0.89) | 0.006 | 15 | 78% | 73% | 74% |
| ELI, % | 0.73 (0.60 − 0.86) | 0.014 | 6.9 | 83% | 56% | 63% |
| TTP, msec | 0.65 (0.48 − 0.83) | 0.448 | 421 | 67% | 73% | 70% |
| ELT, msec | 0.65 (0.50 − 0.79) | 0.576 | 57 | 67% | 64% | 63% |
| PSS, % | 0.61 (0.47 − 0.75) | 1 | -1.3 | 89% | 42% | 53% |
| ESL, % | 0.53 (0.38 − 0.68) | 1 | 0.54 | 89% | 33% | 44% |

**Supplemental Table 5: Receiver-operating characteristics for the detection of patients with large infarcts (quartile with highest infarct size).** AUC, area under the curve; EF, ejection fraction; GLS, global longitudinal peak systolic strain; LPS, longitudinal peak strain; MDI, myocardial dysfunction index; PSI, post-systolic shortening index; PSS, post-systolic shortening; ELI, early systolic lengthening index; ESL, early systolic lengthening; TTP, time-to-peak shortening; ELT, early systolic lengthening time.
